# Venous thromboembolisms and stroke risk in patients with atrial fibrillation: a nationwide cohort study

**DOI:** 10.1093/europace/euaf155

**Published:** 2025-07-29

**Authors:** Eero Jalli, Jussi Jaakkola, Ville Langén, K E Juhani Airaksinen, Olli Halminen, Jukka Putaala, Pirjo Mustonen, Jari Haukka, Juha Hartikainen, Miika Linna, Elis Kouki, Mika Lehto, Konsta Teppo

**Affiliations:** Division of Medicine, Turku University Hospital and University of Turku, Kiinamyllynkatu 4-8, 20521 Turku, Finland; Cardiac Unit, Department of Internal Medicine, Satasairaala, Pori, Finland; Division of Medicine, Turku University Hospital and University of Turku, Kiinamyllynkatu 4-8, 20521 Turku, Finland; Department of Geriatric Medicine, Turku University Hospital and University of Turku, Turku, Finland; Heart Centre, Turku University Hospital and University of Turku, Turku, Finland; Department of Industrial Engineering and Management, Aalto University, Espoo, Finland; Department of Neurology, Helsinki University Hospital and University of Helsinki, Helsinki, Finland; Turku University Hospital and University of Turku, Turku, Finland; Faculty of Medicine, University of Helsinki, Helsinki, Finland; Kuopio University Hospital and Institute of Clinical Medicine, University of Eastern Finland, Kuopio, Finland; Department of Industrial Engineering and Management, Aalto University, Espoo, Finland; University of Eastern Finland, Kuopio, Finland; Faculty of Medicine, University of Helsinki, Helsinki, Finland; Department of Internal Medicine, Jorvi Hospital, HUS Helsinki University Hospital and University of Helsinki, Helsinki, Finland; Division of Medicine, Turku University Hospital and University of Turku, Kiinamyllynkatu 4-8, 20521 Turku, Finland; Heart Centre, Turku University Hospital and University of Turku, Turku, Finland

**Keywords:** Venous thromboembolism, Stroke, Atrial fibrillation, Cohort study

## Abstract

**Aims:**

Little is known about the association of venous thromboembolisms (VTEs) on the risk of ischaemic stroke (IS) in patients with atrial fibrillation (AF). Nevertheless, both pulmonary embolism (PE) and deep venous thromboembolism (DVT) are often included in the calculation of the CHA_2_DS_2_-VASc score, which is used for stroke risk stratification. Therefore, we conducted this nationwide retrospective cohort study to evaluate whether a history of VTE is associated with an increased risk of IS in patients with AF.

**Methods and results:**

The Finnish AntiCoagulation in Atrial Fibrillation (FinACAF) registry-linkage study includes all patients in Finland with incident AF from 2007 to 2018. The IS rates and rate ratios were computed for patients with and without a history of VTE. We identified 271 500 patients with new-onset AF, of whom 4.6% had prior VTE, while 1.9% had a history of PE and 3.1% a history of DVT. The crude incidence of IS was slightly higher in patients with a history of VTE compared to patients without a history of VTE, but after adjusting for baseline factors, VTE was not associated with the rate of IS [adjusted incidence rate ratio with 95% confidence interval for any VTE 1.05 (0.98–1.13), for PE 1.01 (0.91–1.13), and for DVT 1.09 (1.00–1.18)]. There was no temporal change in these associations during the study period.

**Conclusion:**

A history of VTEs was not associated with an increased risk of IS, suggesting that they do not need to be considered in the stroke risk stratification of patients with AF.

## Graphical Abstract

**Graphical Abstract:**
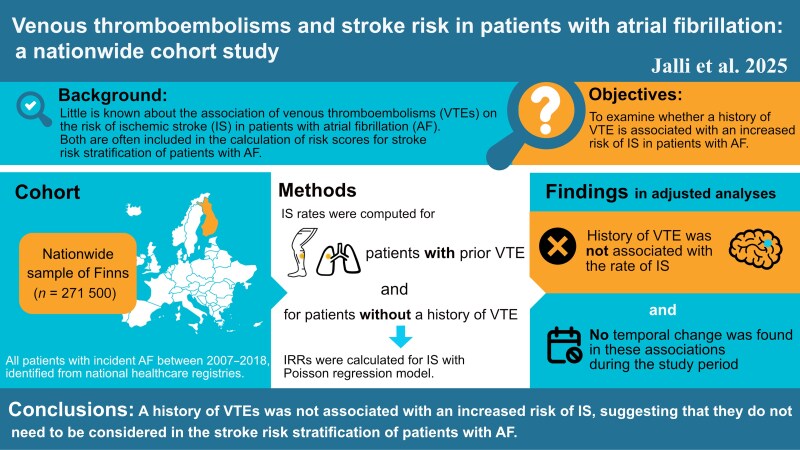

What’s new?

- Previously diagnosed venous thromboembolism (VTE) is not associated with increased risk of stroke in patients with atrial fibrillation (AF).
- The relationship between prior VTE and stroke risk in AF patients has remained consistent over time.
- Findings of this study suggest that there is no need to consider VTEs in stroke risk stratification for patients with AF.

## Introduction

Atrial fibrillation (AF) is the most common sustained cardiac arrhythmia, affecting up to 5.2% of the adult population.^1^ It is a major cause of ischaemic stroke (IS), with the risk of stroke varying considerably among individuals based on their comorbidities and other clinical characteristics.^2,3^ Accurate stratification of stroke risk and identification of individuals who would benefit from oral anticoagulant (OAC) therapy for stroke prevention are essential in managing patients with AF.

Several risk scores have been developed to guide stroke risk stratification and clinical decision-making on OAC therapy. The 2023 American ACC/AHA/ACCP/HRS guidelines on AF management recommend using the CHA_2_DS_2_-VASc score for stroke risk stratification in the absence of other locally calibrated alternatives, whereas the 2024 European Society of Cardiology guidelines now favour the simplified CHA_2_DS_2_-VA score, which does not include the sex component.^4–7^ Interestingly, the definitions of risk factors in these scores have varied over time, and there are some important disparities between the current American and European guidelines. The ACC/AHA/ACCP/HRS guidelines include pulmonary embolism (PE) in the S_2_ category and deep venous thrombosis (DVT) in the V category, whereas the European guidelines restrict the S_2_ category to arterial thromboembolisms and include only atherosclerotic arterial diseases in the V category.^4^

The inclusion of venous thromboembolisms (VTEs) in risk scores has been criticized, as there is no robust evidence supporting that VTE would meaningfully increase the risk for IS in the presence of AF.^8^ Moreover, the past decades have brought significant advancements in the diagnosis and treatment of PE and DVT. The incidence of VTE has been rising, relating to the ageing of the population.^9^ Increased use of computed tomography scans and imaging studies and detection of milder forms of the disease may also be responsible for the growing number of diagnoses. It is also known that while the incidence of PE has increased, PE-related mortality has decreased.^10,11^

Despite the inclusion of VTEs in the CHA_2_DS_2_-VASc score in the American ACC/AHA/ACCP/HRS guideline, clear evidence linking them to an increased stroke risk in patients with AF is lacking. Additionally, considering the changes in the diagnostics and management of both AF and VTE, the association between VTE and IS risk may have also changed. Therefore, we conducted a nationwide retrospective cohort study to examine whether VTEs are associated with an increased risk of IS in patients with AF and whether this association has changed over time.

## Methods

### Study population

The Finnish AntiCoagulation in Atrial Fibrillation (FinACAF) Study (ClinicalTrials identifier: NCT04645537; ENCePP identifier: EUPAS29845) is a nationwide retrospective cohort study that includes all patients documented with AF in Finland from 2004 to 2018.^12^ Patients were identified using all available national healthcare registers, including hospitalizations and outpatient specialist visits (Hilmo), primary healthcare (Avohilmo), and the National Reimbursement Register maintained by the Social Insurance Institute (KELA). The cohort inclusion criterion was the International Classification of Diseases, 10th Revision (ICD-10) diagnosis code I48, encompassing atrial fibrillation and atrial flutter, collectively referred to as AF, recorded between 2004 and 2018. Exclusion criteria were permanent emigration abroad before 31 December 2018 and age under 20 years at the time of AF diagnosis. The present sub-study was conducted within a cohort of patients with incident AF from 2007 to 2018, originally established in earlier studies of the FinACAF cohort and adapted for the purposes of the current study.^13–15^

In this cohort, to include only patients with newly diagnosed AF, a washout period was applied by excluding those with a recorded AF diagnosis during 2004–06, because the medical history of <2 years was considered too short to exclude the presence of a prior AF diagnosis. Additionally, to ensure the exclusion of patients with prior AF, those with a fulfilled OAC prescription during 2004–06 were excluded.

The follow-up was primarily analysed with two separate approaches. In both methods, follow-up started from the initial AF diagnosis. In the first approach, we analysed the overall cohort with the entire follow-up continuing until the first observed IS event, death, or 31 December 2018, whichever came first. In this approach, analyses were adjusted for OAC use. Additionally, since it is the non-anticoagulated IS rate that drives the clinical decision-making of stroke prevention with OACs, in the second approach, we concentrated solely on the follow-up time without OAC therapy. In these analyses, follow-up continued only until the first OAC purchase, the first IS event, death, or 31 December 2018, whichever came first.

### Definition of venous thromboembolism

Previous studies have shown that using hospital-level data alone can underestimate disease prevalence, so we therefore combined available national health registry data to improve the detection of patients with VTE.^16^ Patients were classified as having VTE if they had recorded VTE codes (ICD-10 codes I26 or I80-I82) in the hospital or primary care registers, or VTE reimbursement codes in the National Reimbursement Register. Patients were also analysed separately based on the presence of PE or DVT. Pulmonary embolism was identified using ICD code I26, while DVT was identified using ICD codes I80-I82. A wide variety of deep VTE is included in ICD codes I80-I82, which however corresponds to the definition of the ‘V’ category of the CHA_2_DS_2_-VASc score in the American ACC/AHA/ACCP/HRS guideline.^4^ In the analysis for this study, VTE was not included in the CHA_2_DS_2_-VASc score.

### Outcomes

In patients without prior IS before on the date of the first AF diagnosis, an IS event was considered to occur on the first date of a recorded I63 or I64 ICD-10 diagnosis code in the hospital care register after the cohort entry. In patients with prior IS before or at AF diagnosis, the event was considered to occur on the date of the first new hospitalization with I63 or I64 ICD-10 code as the main diagnosis with at least a 90-day gap from the prior event, which had occurred before AF diagnosis. Only IS diagnoses from the hospital register were included to ensure that the event of interest was truly major and clinically relevant.

### Study ethics

The study protocol was approved by the Ethics Committee of the Medical Faculty of Helsinki University, Helsinki, Finland (nr. 15/2017), and received research permission from the Helsinki University Hospital (HUS/46/2018). Respective permissions were obtained from the Finnish register holders (KELA 138/522/2018, THL 2101/5.05.00/2018, Population Register Centre VRK/1291/2019-3, Statistics Finland TK-53-1713-18/u1281, and Tax Register VH/874/07.01.03/2019). Patients’ personal identification numbers were pseudonymized, and the research group received individualized but unidentifiable data. Informed consent was waived due to the retrospective registry nature of the study. The study conforms to the Declaration of Helsinki as revised in 2013.

### Statistical analyses

We calculated incidence rates and incidence rate ratios (IRRs) for IS using a Poisson regression model. The model employed a Lexis-type data structure, incorporating three time scales: age, follow-up time from AF diagnosis, and calendar year.^17^ This statistical approach accounts for the patients’ increasing age during the relatively long follow-up and aligns outcomes for each calendar year period. Age was categorized into 10-year intervals, and the 12-year observation period was divided into 4-year intervals. Both age and calendar year were analysed as categorical variables. Adjusted IRRs accounted for the following variables: age, calendar year period, sex, hypertension, diabetes, prior IS, vascular disease, dyslipidaemia, prior bleeding, alcohol use disorder, renal failure, liver cirrhosis or failure, dementia, income level (divided into tertiles), and OAC use. The OAC use was treated as a time-varying variable, initiated by the first purchase of an OAC and continuing until 120 days after the last drug purchase. In Finland, individuals are reimbursed for up to 90 days of medication per purchase, and the registry lacks data on the exact quantity dispensed. We therefore assumed each purchase covered 90 days. We used the 120-day interval to encompass this 90-day period and an additional 30-day grace period to account for potential stockpiling and variations in warfarin dosing. When only time without OAC therapy was investigated, OAC use was not included in the adjusted analyses. Subsequently, the models were fitted with an interaction term between calendar year period and VTE to assess changes in the impact of VTE on IS over time. Additionally, separate sensitivity analyses were performed to assess the association between VTEs and IS among patients without prior stroke before the diagnosis of AF. Baseline variables were compared using the χ^2^ test, Student’s *t*-test, and one-way analysis of variance. Statistical significance was assessed with the 95% confidence intervals (CIs) of the IRRs. Statistical analyses were conducted using IBM SPSS Statistics software version 28.0 (SPSS, Inc., Chicago, IL, USA) and R version 4.0.5 (R Core Team, Vienna, Austria; https://www.R-project.org).

## Results

We identified 271 500 patients with new-onset AF, of whom 4.6% had prior VTE, while 1.9% had a history of PE and 3.1% a history of DVT. The VTE group had a higher proportion of women (58.1% vs. 48.7%), was generally older (76.1 vs. 72.7), and had lower income, a higher burden of comorbidities, and a higher mean CHA_2_DS_2_-VASc score (4.0 vs. 3.4) compared to individuals without VTE (*Table 1*). When considering PE and DVT separately, their characteristics of the study cohort were largely similar, although patients with PE had a somewhat greater burden of comorbidities than those with DVT (*Table 1*).

**Table 1:** Baseline characteristics of the study cohort according to the history of VTE

	No venous thromboembolism	Any venous thromboembolism	Deep venous thrombosis	Pulmonary embolism
	*n* = 258 897	*n* = 12 603	*n* = 8434	*n* = 5076
Demographics				
Mean age, years	72.7 (12.9)	76.1 (11.2)	76.0 (11.3)	76.2 (11.0)
Female sex	48.7	58.1	59.0	55.4
Income tertiles				
1st (lowest)	33.7	38.8	37.7	39.9
2nd	32.9	34.3	34.3	34.3
3rd (highest)	33.4	26.9	28.0	25.8
Comorbidities				
Any vascular disease	28.1	36.7	35.0	39.6
Prior MI	8.5	11.3	10.4	12.5
Diabetes	21.9	24.9	24.6	25.1
Dyslipidaemia	48.7	53.8	54.4	52.7
Hypertension	75.8	81.1	81.1	80.9
Heart failure	17.2	28.7	24.8	35.7
Prior IS	11.2	13.4	13.6	12.8
Abnormal liver function	0.4	0.8	0.9	0.6
Abnormal renal function	3.8	8.1	7.6	9.1
Alcohol use disorder	3.8	4.2	4.1	4.4
Dementia	4.9	7.4	7.5	7.0
Prior bleeding	10.4	18.1	17.7	18.8
Risk scores				
Mean modified HAS-BLED score	2.1 (1.0)	2.4 (1.0)	2.4 (1.0)	2.4 (1.0)
Mean CHA_2_DS_2_-VASc score	3.4 (1.9)	4.1 (1.8)	4.0 (1.8)	4.1 (1.8)
Mean CHA_2_DS_2_-VA score	2.9 (1.7)	3.5 (1.7)	3.4 (1.7)	3.6 (1.7)

Values denote proportions (%) or means (standard deviation). All differences between any VTE and no thromboembolism *P* < 0.001, except alcohol use disorder (*P* = 0.057).

MI, myocardial infarction; CHA_2_DS_2_-VASc score, congestive heart failure (1 point), hypertension (1 point), age ≥ 75 years (2 points), diabetes (1 point), history of stroke or TIA (2 points), vascular disease (1 point), age 65–74 years (1 point), sex category (female) (1 point); CHA_2_DS_2_-VA score same as CHA_2_DS_2_-VASc score but without sex category; IS, ischaemic stroke; modified HAS-BLED score, hypertension (1 point), abnormal renal or liver function (1 point each), prior stroke (1 point), bleeding history (1 point), age > 65 years (1 point), alcohol abuse (1 point), concomitant antiplatelet/NSAIDs (1 point) (no labile INR, max score 8).

**Figure 1:**
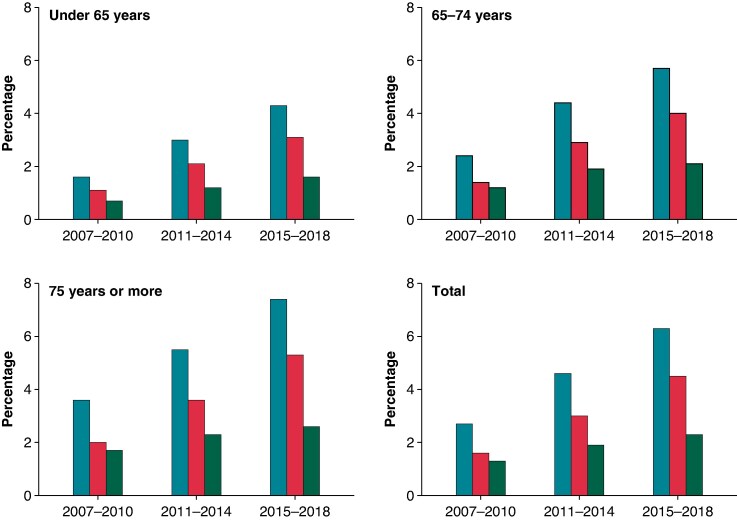
Prevalence of VTE history before AF diagnosis according to age in the study cohort.

The prevalence of prior VTE before AF diagnosis in the cohort increased during the study period from 2.7% during 2007–10 to 6.3% during 2015–18. This increase was primarily driven by a rise in the prevalence of DVT, which increased from 1.6% during 2007–10 to 4.5% during 2015–18. The prevalence of PE among AF patients exhibited a smaller increase from 1.3% during 2007–10 to 2.3% during 2015–18. The observed increases in prevalence were consistent across all age categories (*Figure 1*; Supplementary material online, *Table S1*).

The crude IS incidence rate decreased steadily from 2007–10 to 2015–18 both in patients with and without a history of VTE. However, the IS rate remained consistently higher in patients with a prior VTE (see Supplementary material online, *Figure S1*). Similar decreasing IS trends were observed when analysing patients with a history of PE or DVT alone (see Supplementary material online, *Figures S2* and *S3*).

**Figure 2:**
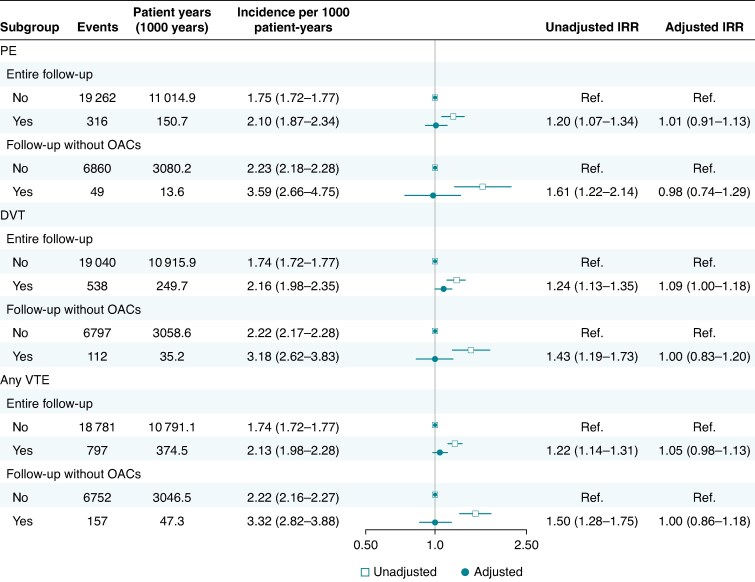
Incidence of IS in patients with and without VTE from 2007 to 2018, with entire follow-up and follow-up without OAC use. 95% CIs in parenthesis. IRR, incidence rate ratio; PE, pulmonary embolism; DVT, deep venous thromboembolism; VTE, venous thromboembolism.

The cumulative incidence of IS following an AF diagnosis increased progressively in both patients with and without a history of VTE (see Supplementary material online, *Figure S4*). The crude incidence of IS was higher in patients with VTEs, compared to those without a history of VTE (*Figure 2*).

In the analyses covering the entire follow-up, VTE was not independently associated with the rate of IS after adjusting for confounding factors, adjusted IRR 1.05 (95% CI: 0.98–1.13). When assessing PE and DVT separately, PE was not associated with IS, while DVT showed a marginally statistically significant association with IS [adjusted IRRs with 95% CIs 1.01 (0.91–1.13) and 1.09 (1.00–1.18), respectively] (*Figure 2*). Moreover, when the analysis was restricted to patients with a low to moderate risk of stroke (CHA_2_DS_2_-VA score of 0 or 1 according to the European AF guidelines), neither VTE, PE, nor DVT individually was associated with IS (see Supplementary material online, *Figure S5*). Results were also consistent in the analyses restricted to patients without a prior IS [adjusted IRR: 1.01 (0.90–1.15), 1.09 (0.99–1.20), and 1.05 (0.97–1.13) for PE, DVT, and any VTE, respectively]. When considering only time without OAC treatment, neither VTE nor PE and DVT individually were associated with IS in the adjusted analyses (*Figure 2*).

**Figure 3:**
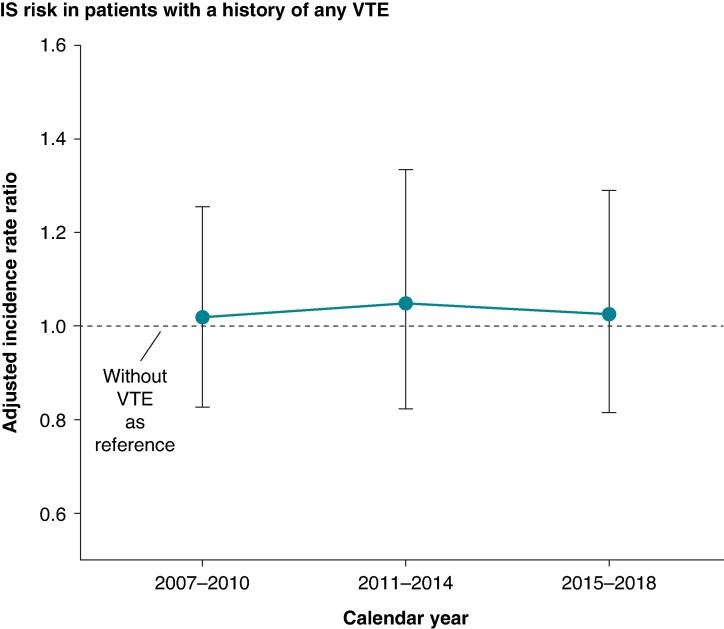
Adjusted IRR with 95% CIs for IS, comparing patients with and without any VTE. The broken line represents patients without VTE. *P*-value for interaction with calendar year period 0.92.

The association between VTE and IS remained stable across the study period, and no statistically significant change was observed in this association. The results were similar when analysing patients with a history of PE or DVT alone (*P*-values for interaction of VTE, PE, and DVT with calendar year periods: 0.92, 0.69, and 0.67, respectively) (*Figure 3*; Supplementary material online, *Figures S6* and *S7*).

## Discussion

This nationwide retrospective cohort study examining the relationship between a history of VTE and IS risk in patients with AF during 2007–18 demonstrated that VTEs are not associated with the risk of IS, and there has been no change in this association during the study period. These findings can have important implications on the use of stroke risk scores and patient selection for OAC therapy.

The disparities in stroke risk stratification scores recommended by American and European guidelines on AF management highlight uncertainty regarding the inclusion of VTEs in stroke risk assessment and decision-making on OAC therapy in patients with AF.^4,5,8^ Indeed, previous studies relying on only hospital-level data have suggested that AF patients with a history of VTE carry a similar risk of IS compared to those without a VTE history.^18,19^ The current study, utilizing comprehensive nationwide data across all levels of care, provides needed robust evidence on this topic, demonstrating that neither VTEs in general, nor PE or DVT individually, meaningfully increase the risk of IS in patients with AF. Although marginal statistical significance was observed in the association between DVT and IS risk in the adjusted analyses covering also time with OAC use, this was no longer the case when considering only the time without OAC, which is more relevant in the context of AF and stroke prevention. Moreover, none of the VTE categories were associated with IS in patients with low to moderate risk, in whom an additional score based on VTE could affect decision-making regarding OAC therapy. Overall, our findings suggest that there is no need to consider VTEs in stroke risk stratification for patients with AF.

The prevalence of VTE history increased during the study period, which aligns with previous literature on increasing comorbidities in patients with AF.^20^ Similar trends regarding VTEs have also been observed at the population level, possibly driven by improved access to computed tomography scans and other imaging techniques, which enable the diagnosis of milder forms of VTE.^10,11,21,22^ Indeed, as the number of patients with a history of VTE continues to rise, we are increasingly faced with the decision of whether to initiate OAC therapy for patients with incident AF and a prior VTE. As mentioned earlier, the American ACC/AHA/ACCP/HRS guidelines for AF include previous PE or DVT in the widely used CHA_2_DS_2_-VASc risk score, thus favouring the initiation of OAC treatment for patients with prior VTEs.^4^ Our findings suggest that, when focusing specifically on stroke risk stratification and prevention, the cornerstone in the management of patients with AF, history of VTE may not need to be considered. Importantly, VTEs were not associated with an increased risk of stroke in AF patients at low to moderate stroke risk—the group in which potential assignment of an additional point for VTE would most affect treatment decisions and might prompt initiation of oral anticoagulation. However, the American guidelines may contain some pragmatism in terms of preventing also future VTEs, and some validation studies of the CHA_2_DS_2_-VASc score have even considered PE as an outcome event, while others have specifically focused on estimating stroke rates at different score levels.^23–25^ It is also notable that AF and history of VTE are two distinct, well-established risk factors for thromboembolism—whether arterial or venous. In borderline cases for initiating OAC therapy, clinicians often rely on individual judgment and may choose to initiate OAC in patients who present with two separate but relatively weak indications.

Over recent decades, substantial advances have been made in AF treatment—particularly in stroke prevention, the identification of risk factors, and the understanding of AF-related stroke epidemiology and pathophysiology.^26^ Despite the significant changes in the diagnostics and treatment of both VTE and AF over recent decades, our study shows that there have not been any significant changes in the association between the risk of IS and a history of prior VTE. No statistically significant changes were observed, also with the DVT or PE groups. This finding further supports the conclusion that a history of VTE does not meaningfully associate with an increased risk of IS in AF patients.

The retrospective registry-based design of our study introduces some limitations that should be considered. First, our results may be affected by information bias due to inaccuracy of registry data such as missing or inaccurate reporting of diagnostic codes of PE or DVT. The registry data also lacked detailed information on whether the VTEs were provoked, such as cancer-associated, and whether they were symptomatic or asymptomatic.^27,28^ We also lacked information on the specific type of AF and how AF was detected—a factor that can impact the sensitivity and accuracy of AF diagnosis.^29^ To improve accuracy, we linked data from registries of different levels of care. Second, the cohort construction process may introduce some selection bias. Third, due to the retrospective nature of our study, our results represent associations and not necessarily causal relationships. Finally, despite adjusting for multiple variables, some residual confounding may remain in the results. Nevertheless, our study has the advantages of a nationwide coverage through all national healthcare registries, encompassing uniquely all patients with incident AF in Finland from all levels of care. The utilization of the well-validated hospital care register enhances the reliability of the observed IS outcomes, and the medication information is derived from complete nationwide pharmacy data on redeemed prescriptions.^30^

In conclusion, this nationwide study covering all levels of care found no association between a history of VTE and an increased risk of IS in patients with AF. Our results suggest that there is no need to consider prior VTEs in the decision-making process of stroke preventive therapies in patients with AF.

## Supplementary Material

euaf155_Supplementary_Data

## Data Availability

Because of the sensitive nature of the data collected for this study, requests to access the dataset from qualified researchers trained in human subject confidentiality protocols may be sent to the Finnish national register holders (KELA, Finnish Institute for Health and Welfare, Population Register Center, and Tax Register) through Findata (https://findata.fi/en/). In the interest of research transparency and reproducibility, we have made all analysis code used in this study publicly available via GitHub and permanently archived on Zenodo under DOI: 10.5281/zenodo.15065420. The code can be accessed through the DOI search portal at https://www.doi.org or directly via https://zenodo.org/records/15065421.
