# Supplementary material for: Venous thromboembolisms and stroke risk in patients with atrial fibrillation: a nationwide cohort study
Source: Europace. 2025 Jul 29;27(8):euaf155. doi: 10.1093/europace/euaf155 (PMC12365899; doi:10.1093/europace/euaf155)
Supplement: euaf155_Supplementary_Data [file euaf155_supplementary_data.docx]

**Supplemental Material**

1. Supplementary Table 1. Prevalence of venous thromboembolism according to age in the study cohort
2. Supplementary Figure 1. Trends in crude ischemic stroke rate in patients with and without prior venous thromboembolism (VTE) with 95% confidence intervals between 2007 and 2018 in the entire study population.
3. Supplementary Figure 2. Trends in crude ischemic stroke rate in patients with and without prior pulmonary embolism (PE) with 95% confidence intervals between 2007 and 2018 in the entire study population.
4. Supplementary Figure 3. Trends in crude ischemic stroke rate in patients with and without prior deep venous thromboembolism (DVT) with 95% confidence intervals between 2007 and 2018 in the entire study population.
5. Supplementary Figure 4. Cumulative incidence of ischemic stroke in patients with and without a history of prior VTE within the study cohort.
6. Supplementary Figure 5. Incidence of ischemic stroke in patients with low to moderate risk of stroke with and without a history of venous thromboembolism from 2007 to 2018, with follow-up adjusted for oral anticoagulant use.
7. Supplementary Figure 6. Adjusted incidence rate ratio for ischemic stroke (IS), comparing patients with and without prior pulmonary embolism (PE).
8. Supplementary Figure 7. Adjusted incidence rate ratio for ischemic stroke (IS), comparing patients with and without prior deep venous thromboembolism (DVT).

**Supplementary Table 1.** History of venous thromboembolism at diagnosis of AF according to age in the study cohort.

|  | **2007-2010** | **2011-2014** | **2015-2018** |
| --- | --- | --- | --- |
| **Under 65 years** | | | |
| Any venous thromboembolism | 1.6 (376) | 3.0 (690) | 4.3 (873) |
| Pulmonary embolism | 0.7 (158) | 1.2 (278) | 1.6 (329) |
| Deep venous thrombosis | 1.1 (245) | 2.1 (477) | 3.1 (627) |
| **From 65 to 74 years** | | | |
| Any venous thromboembolism | 2.4 (490) | 4.4 (1132) | 5.7 (1624) |
| Pulmonary embolism | 1.2 (248) | 1.9 (489) | 2.1 (586) |
| Deep venous thrombosis | 1.4 (274) | 2.9 (734) | 4.0 (1140) |
| **75 years or more** | | | |
| Any venous thromboembolism | 3.6 (1297) | 5.5 (2560) | 7.4 (3561) |
| Pulmonary embolism | 1.7 (629) | 2.3 (1082) | 2.6 (1277) |
| Deep venous thrombosis | 2.0 (745) | 3.6 (1652) | 5.3 (2540) |
| **Total** | | | |
| Any venous thromboembolism | 2.7 (2163) | 4.6 (4382) | 6.3 (6058) |
| Pulmonary embolism | 1.3 (1035) | 1.9 (1849) | 2.3 (2192) |
| Deep venous thrombosis | 1.6 (1264) | 3.0 (2863) | 4.5 (4307) |
| Values denote proportions of all patients with incident AF and count in parenthesis. P-values for differences within age groups all <0.001. | | | |


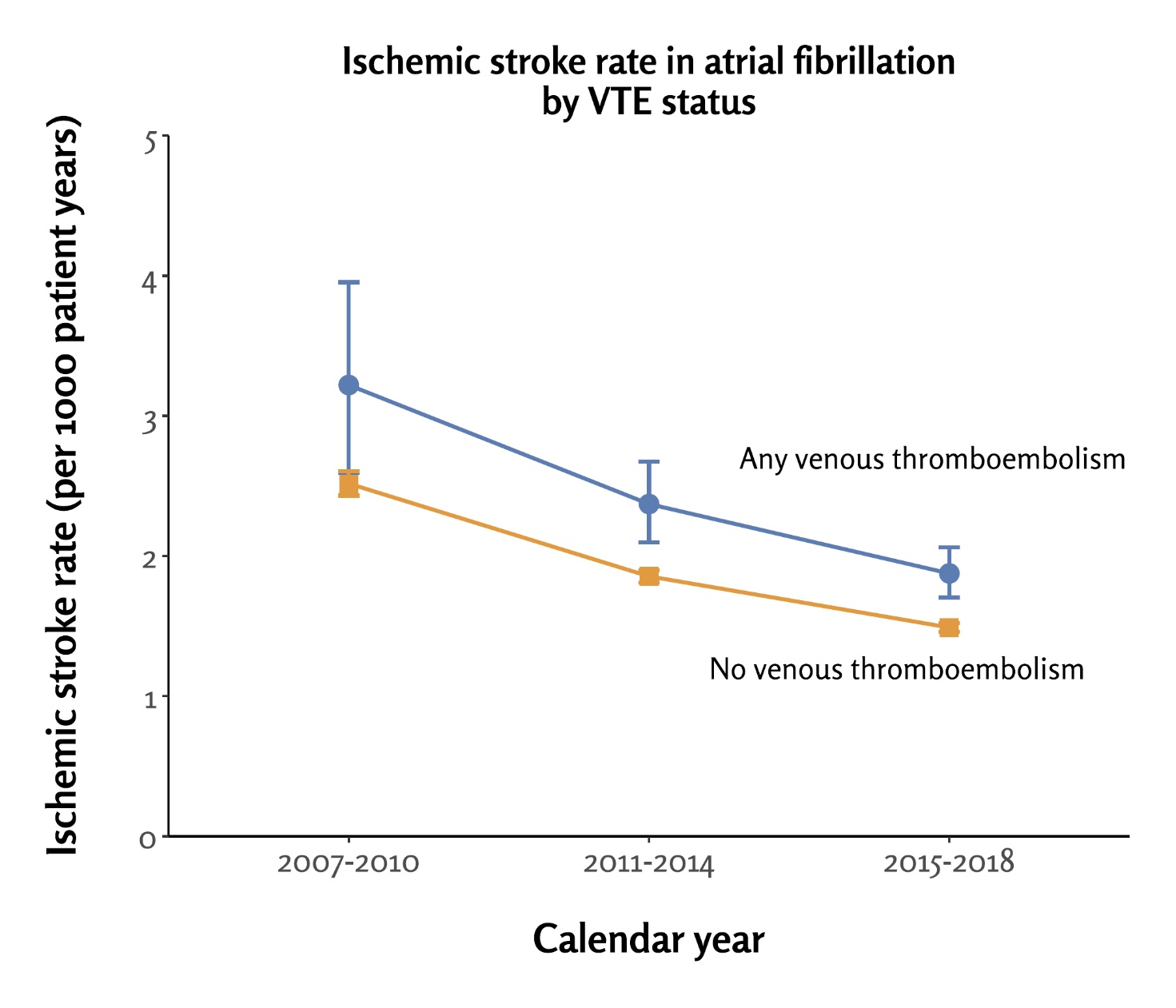


**Supplementary Figure 1.** Trends in crude ischemic stroke rate in patients with and without prior venous thromboembolism (VTE) with 95% confidence intervals between 2007 and 2018 in the entire study population.


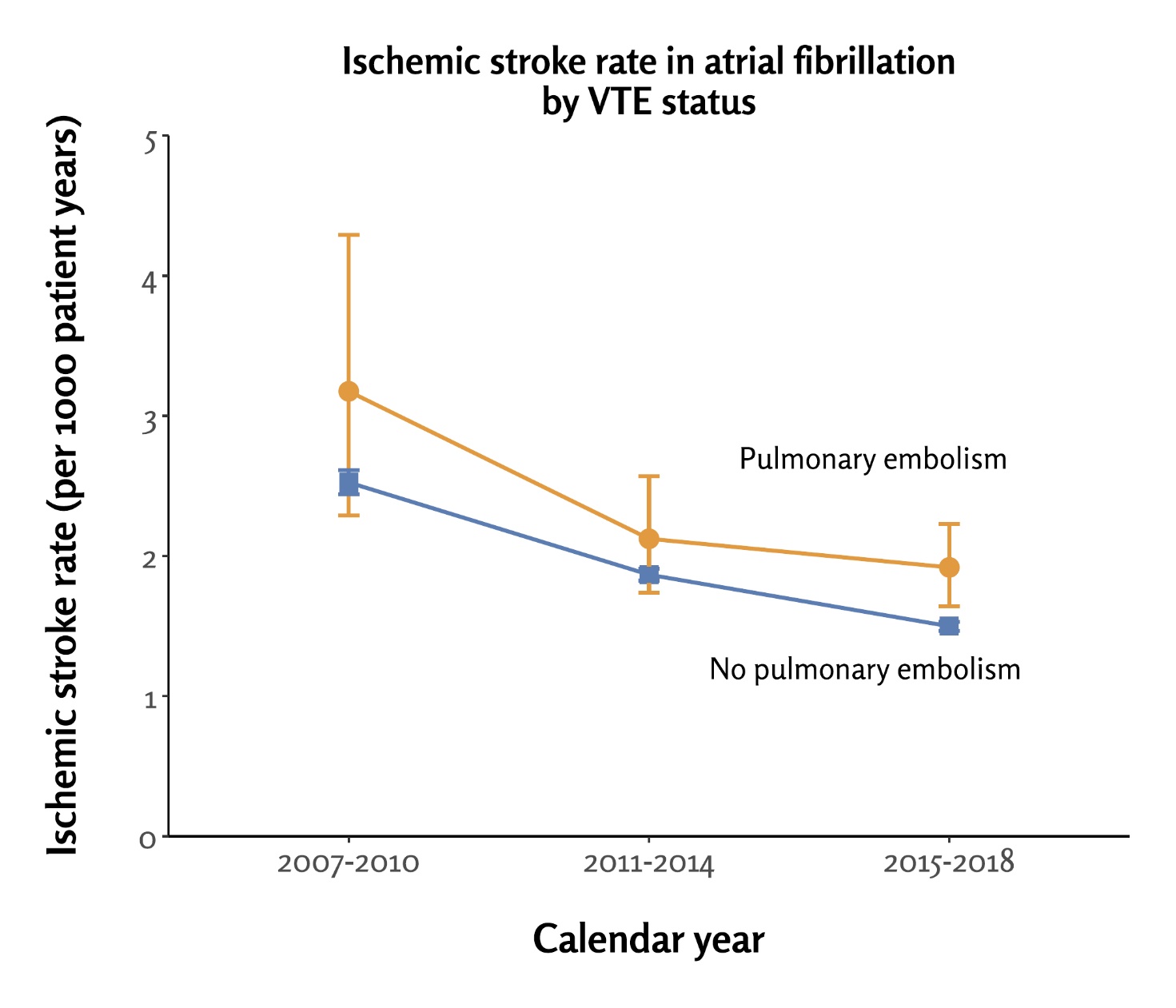


**Supplementary Figure 2.** Trends in crude ischemic stroke rate in patients with and without prior pulmonary embolism (PE) with 95% confidence intervals between 2007 and 2018 in the entire study population.


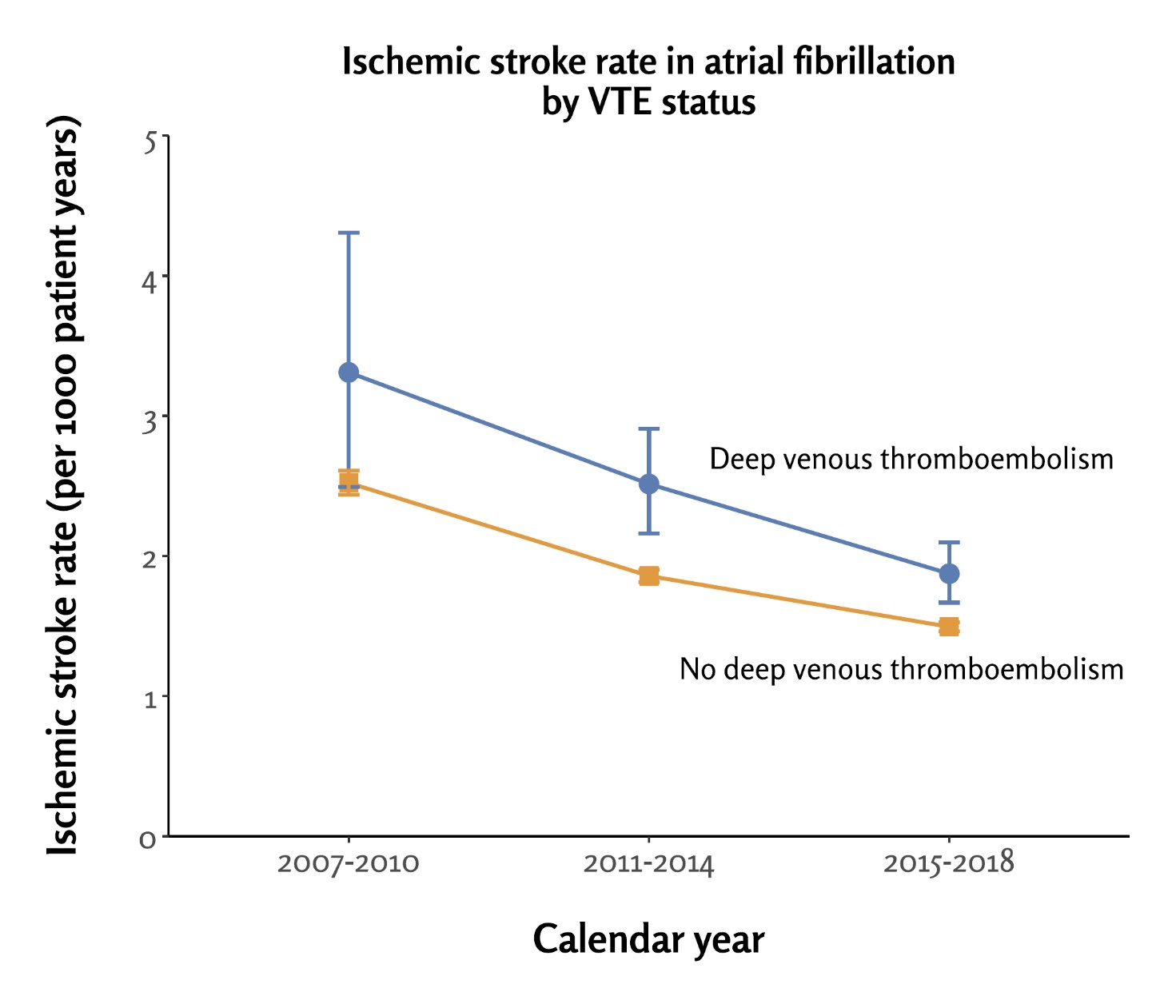


**Supplementary Figure 3.** Trends in crude ischemic stroke rate in patients with and without prior deep venous thromboembolism (DVT) with 95% confidence intervals between 2007 and 2018 in the entire study population.


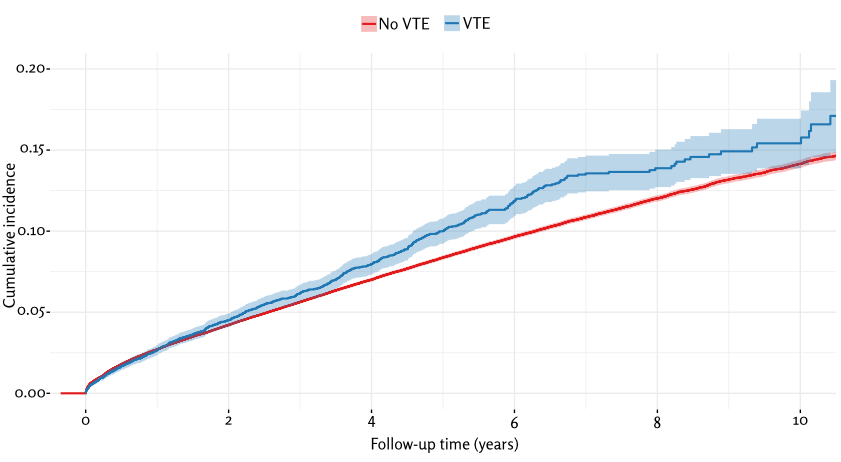


**Supplementary Figure 4.** Cumulative incidence of ischemic stroke in patients with and without a history of prior VTE within the study cohort.


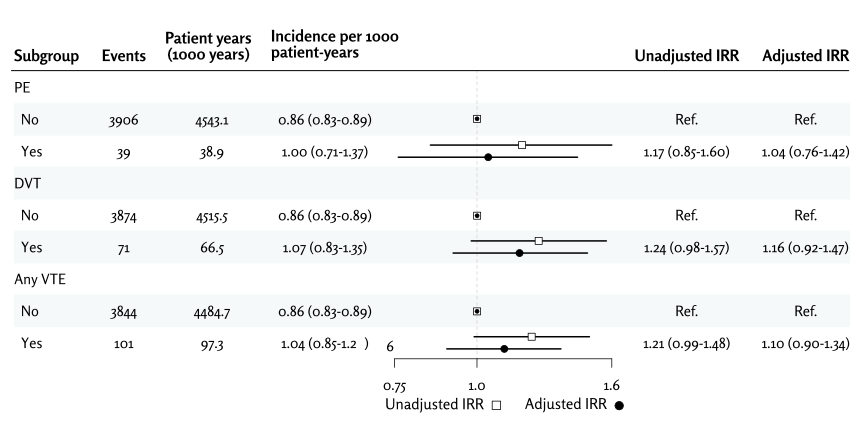


**Supplementary Figure 5.** Incidence of ischemic stroke in patients with low to moderate risk of stroke with and without a history of venous thromboembolism from 2007 to 2018, with follow-up adjusted for oral anticoagulant use. 95% confidence intervals in parenthesis. IRR, incidence rate ratio; PE, pulmonary embolism; DVT, deep venous thromboembolism; VTE, venous thromboembolism.


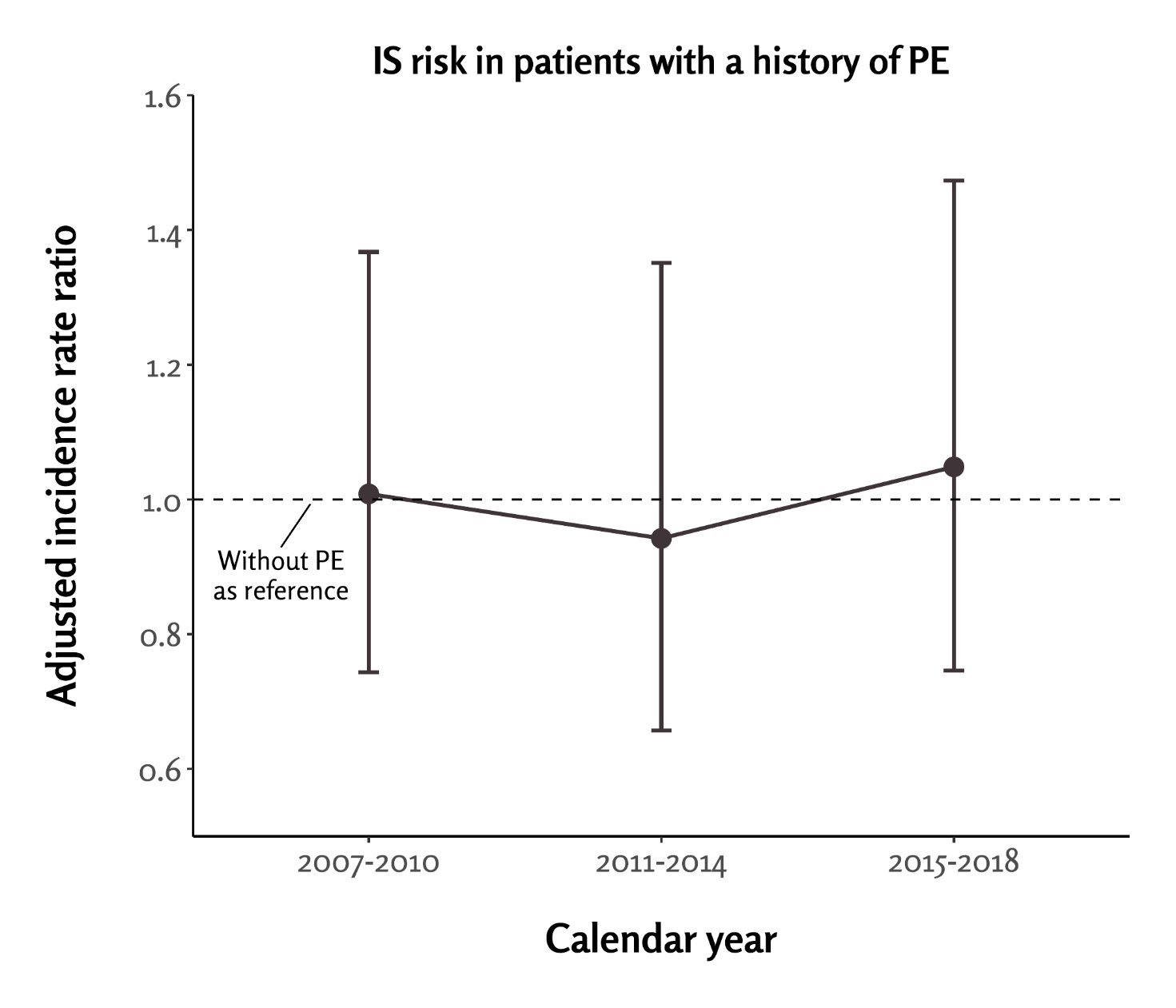


**Supplementary Figure 6.** Adjusted incidence rate ratio with 95% confidence intervals for ischemic stroke (IS), comparing patients with and without prior pulmonary embolism (PE). The broken line represents patients without venous thromboembolism. P-value for interaction with calendar year period 0.69.


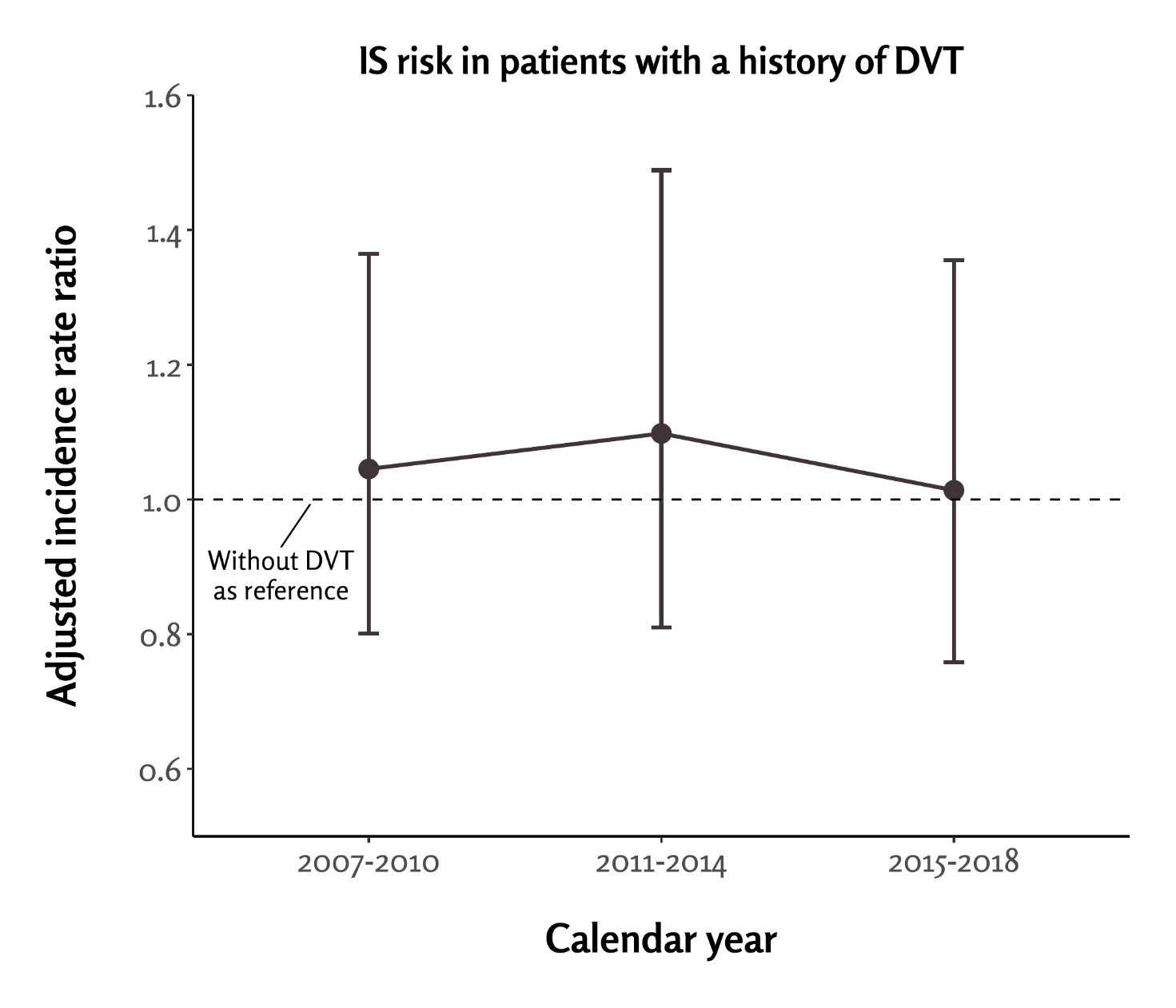


**Supplementary Figure 7.** Adjusted incidence rate ratio with 95% confidence intervals for ischemic stroke (IS), comparing patients with and without prior deep venous thromboembolism (DVT). The broken line represents patients without venous thromboembolism. P-value for interaction with calendar year period 0.67.
